# A Prospective Study of Red and Processed Meat Intake in Relation to Cancer Risk

**DOI:** 10.1371/journal.pmed.0040325

**Published:** 2007-12-11

**Authors:** Amanda J Cross, Michael F Leitzmann, Mitchell H Gail, Albert R Hollenbeck, Arthur Schatzkin, Rashmi Sinha

**Affiliations:** 1 Nutritional Epidemiology Branch, Division of Cancer Epidemiology and Genetics, National Cancer Institute, National Institutes of Health, Department of Health and Human Services, Rockville, Maryland, United States of America; 2 Biostatistics Branch, Division of Cancer Epidemiology and Genetics, National Cancer Institute, National Institutes of Health, Department of Health and Human Services, Rockville, Maryland, United States of America; 3 AARP, Washington DC, United States of America; McGill University, Canada

## Abstract

**Background:**

Red meat and processed meat have been associated with carcinogenesis at several anatomic sites, but no prospective study has examined meat intake in relation to a range of malignancies. We investigated whether red or processed meat intake increases cancer risk at a variety of sites.

**Methods and Findings:**

The National Institutes of Health (NIH)-AARP (formerly the American Association for Retired Persons) Diet and Health Study is a cohort of approximately 500,000 people aged 50–71 y at baseline (1995–1996). Meat intake was estimated from a food frequency questionnaire administered at baseline. Cox proportional hazards regression was used to estimate hazard ratios and 95% confidence intervals within quintiles of red and processed meat intake. During up to 8.2 y of follow-up, 53,396 incident cancers were ascertained. Statistically significant elevated risks (ranging from 20% to 60%) were evident for esophageal, colorectal, liver, and lung cancer, comparing individuals in the highest with those in the lowest quintile of red meat intake. Furthermore, individuals in the highest quintile of processed meat intake had a 20% elevated risk for colorectal and a 16% elevated risk for lung cancer.

**Conclusions:**

Both red and processed meat intakes were positively associated with cancers of the colorectum and lung; furthermore, red meat intake was associated with an elevated risk for cancers of the esophagus and liver.

## Abstract 2

Using data from a large cohort study, Amanda Cross and colleagues found that both red and processed meat intakes were positively associated with cancers of the colorectum and lung.

## Editors' Summary

**Background.:**

Every year, there are more than 10 million new cases of cancer around the world. These cases are not spread evenly across the globe. The annual incidence of cancer (the number of new cases divided by the population size) and the type of cancer most commonly diagnosed varies widely among countries. Much of the global variation in cancer incidence and type is thought to be due to environmental influences. These include exposure to agents in the air or water that cause cancer, and lifestyle factors such as smoking and diet. Researchers identify environmental factors that affect cancer risk by measuring the exposure of a large number of individuals to a specific environmental factor and then monitoring these people for several years to see who develops cancer. The hope is that by identifying the environmental factors that cause or prevent cancer, the global burden of cancer can be reduced.

**Why Was This Study Done?:**

Diet is thought to influence the incidence of several cancers but it is very difficult to unravel which aspects of diet are important. Being overweight, for example, is strongly associated with an increased risk of developing several types of cancer, but the evidence that the intake of red meat (beef, pork, and lamb) and of processed meat (for example, bacon, ham, and sausages) is linked to cancer risk is much weaker. Although several studies have linked a high intake of red meat and processed meat to an increased risk of colorectal cancer (the colon is the large bowel; the rectum is the final few inches of the large bowel before the anus), whether this aspect of diet affects the risk of other types of cancer is unclear. In this prospective study, the researchers have examined the association between meat intake and the incidence of a wide range of cancers.

**What Did the Researchers Do and Find?:**

In 1995–1996, nearly half a million US men and women aged 50–71 y joined the NIH-AARP Diet and Health Study. The participants in this study—none of whom had had cancer previously—completed a questionnaire about their dietary habits over the previous year and provided other personal information such as their age, weight, and smoking history. The researchers used these data and information from state cancer registries to look for associations between the intake of red and processed meat and the incidence of various cancers. They found that people whose red meat intake was in the top fifth of the range of intakes recorded in the study (the highest quintile of consumption) had an increased risk of developing colorectal, liver, lung, and esophageal cancer when compared with people in the lowest quintile of consumption. People in the highest quintile of processed meat intake had an increased risk of developing colorectal and lung cancer. The incidences of other cancers were largely unaffected by meat intake.

**What Do These Findings Mean?:**

These findings provide strong evidence that people who eat a lot of red and processed meats have greater risk of developing colorectal and lung cancer than do people who eat small quantities. They also indicate that a high red meat intake is associated with an increased risk of esophageal and liver cancer, and that one in ten colorectal and one in ten lung cancers could be avoided if people reduced their red and processed meat intake to the lowest quintile. However, although the researchers allowed for factors such as smoking history that might have affected cancer incidences, some of the effects they ascribe to meat intake might be caused by other lifestyle factors. Furthermore, because the study's definitions of red meat and processed meat overlapped—bacon and ham, for example, were included in both categories—exactly which type of meat is related to cancer remains unclear. Finally, most of the study participants were non-Hispanic white, so these findings may not apply to people with different genetic backgrounds. Nevertheless, they add to the evidence that suggests that decreased consumption of red and processed meats could reduce the incidence of several types of cancer.

**Additional Information.:**

Please access these Web sites via the online version of this summary at http://dx.doi.org/10.1371/journal.pmed.0040325.

- The American Cancer Society provides answers to common questions about diet and cancer
- Information is available from the charity Cancer Research UK about diet, healthy eating, and cancer
- The American Institute for Cancer Research also provides information on diet and cancer.
- The NIH-AARP Diet and Health Study presents information on the impact of diet and lifestyle factors on risk of cancer
- The US National Cancer Institute provides information about the kind of food questionnaire used in this study

## Introduction

Much of the global variation in cancer incidence has been attributed to environmental influences, including dietary preferences. Not only does meat intake vary substantially around the world, but diets high in red or processed meats have been associated with carcinogenesis at a variety of anatomic sites. The evidence to support a positive association between meat intake and carcinogenesis is based on an assortment of research ranging from laboratory studies [1–3] to observational epidemiology [4–8].

Thus far, the majority of epidemiologic meat research has focused on the more prevalent cancers, particularly colorectal cancer. The most recent meta-analysis of prospective studies of meat and colorectal cancer reported significantly elevated summary relative risks (RRs) for both red meat (RR = 1.28; 95% confidence interval (CI) = 1.15–1.42) and processed meat (RR = 1.20; 95% CI = 1.11–1.31) in the highest versus lowest category of intake [9]. To date, findings for other major cancers—such as prostate, breast, lung, and pancreatic cancer—are less consistent. Prospective data for rarer cancers are sparse, and most epidemiologic studies of less–commonly occurring cancers are restricted to case-control studies, for which investigations of dietary associations are difficult, because of the potential for recall bias [10].

We prospectively investigated red and processed meat intake in relation to cancer incidence in a cohort of approximately half a million men and women enrolled in the National Institutes of Health (NIH)-AARP (formerly known as the American Association for Retired Persons) Diet and Health Study. This study's large size facilitated the investigation of comparatively rare cancers that have not yet been prospectively investigated.

## Materials and Methods

### Study Population

The NIH-AARP Diet and Health Study is a large prospective cohort of men and women, aged 50–71 y, from six states in the United States (California, Florida, Louisiana, New Jersey, North Carolina, and Pennsylvania) and two metropolitan areas (Atlanta, Georgia; and Detroit, Michigan). Recruitment began in 1995 when a self-administered questionnaire eliciting information on demographic and lifestyle characteristics, including dietary habits, was mailed to 3.5 million members of AARP; further details of the design of the study have been described in detail elsewhere [11,12]. The NIH-AARP Diet and Health Study was approved by the Special Studies Institutional Review Board of the US National Cancer Institute, and written informed consent was obtained from all participants by virtue of completing the baseline questionnaire.

### Dietary Assessment

A 124-item food frequency questionnaire (FFQ), based on the Diet History Questionnaire (http://riskfactor.cancer.gov/DHQ/forms/files/shared/dhq1.2002.sample.pdf), developed at the National Cancer Institute, was completed at baseline. The FFQ assessed the usual frequency of consumption and portion size information (based on three categories of <25th, 25th–75th, and >75th percentile of intake from national dietary data) of foods and drinks over the previous 12 mo. Portion sizes and daily nutrient intakes were calculated from the 1994–1996 US Department of Agriculture's Continuing Survey of Food Intake by Individuals [13]. The FFQ compared favorably to other FFQs [14], and was also calibrated within this study population against two 24-h recall diaries [12]. Red meat intake was calculated from the frequency of consumption and portion size information of all types of beef, pork, and lamb; this included bacon, beef, cold cuts, ham, hamburger, hot dogs, liver, pork, sausage, and steak. The processed meat variable included bacon, red meat sausage, poultry sausage, luncheon meats (red and white meat), cold cuts (red and white meat), ham, regular hot dogs, and low-fat hot dogs made from poultry. The meat variables also included meats added to complex food mixtures, such as pizza, chili, lasagna, and stew.

### Cohort Follow-Up and Case Ascertainment

Cohort members are followed annually for change of address using the US Postal Service, and vital status is ascertained by annual linkage to the US Social Security Administration Death Master File. Follow-up for these analyses was calculated from baseline (1995–1996) until censoring at the end of 2003, or when the participant moved out of one of the eight study areas, had a cancer diagnosis, or died, whichever came first. Cancer cases were identified by linkage to state cancer registries and the National Death Index Plus. The eight state cancer registry databases are estimated to be 95% complete within 2 y of cancer incidence and are certified by the North American Association of Central Cancer Registries for meeting the highest standard of data quality [11]. Cancer diagnoses contributed to the incidence of the tumor site of the first diagnosis only and not subsequent diagnoses for additional sites. The cancer endpoints were defined by anatomic site and histologic code of the International Classification of Diseases for Oncology (ICD-0–3) [15]. Advanced prostate cancer may have a distinct etiology; therefore, we used the Tumor-Node-Metastasis classification system [16], and defined those with clinical or pathologic stage T3, T4, or N1 or M1, as well as individuals who died of prostate cancer, to have advanced disease. Results are presented for cancer sites with at least 60 cases within each sex.

### Statistical Analysis

A total of 567,169 persons returned the baseline questionnaire and were available for analysis. We excluded individuals with duplicate records (*n* = 179), those who died before returning the baseline questionnaire (*n* = 261), those who had zero person years of follow-up (*n* = 32), those who moved out of the eight study areas before returning the questionnaire (*n* = 269), those who requested to be withdrawn from the study (*n* = 1), those whose questionnaire was filled in by someone else on their behalf (*n* = 15,765), those who had prevalent cancer (as noted by cancer registry or self-report) at baseline (*n* = 51,212) or end-stage renal disease (*n* = 997), and those with extreme daily total energy intake (*n* = 4,417), defined as more than two inter-quartile ranges above the 75th or below the 25th percentile on the logarithmic scale. After all exclusions, our analytic cohort consisted of 494,036 persons (294,724 men and 199,312 women).

Hazard ratios (HRs) and 95% CIs were estimated using Cox proportional hazards regression with time since entry (person years) as the underlying time metric. Analyses using age as the underlying time metric yielded almost identical HRs. We controlled for age as a continuous variable in the Cox model; inclusion of a quadratic term for age did not improve the fit of the model, nor did it modify the HRs appreciably. Multivariate HRs are reported within quintiles, using the lowest quintile as the referent category, adjusted for the covariates listed in the tables. Missing data were minimal for this study; where appropriate—i.e., for smoking history and body mass index (BMI)—we created “missing” categories, but for others, such as family history of cancer, we set individuals missing this data to zero: no family history. Saturated fat intake and menopausal hormone therapy were investigated as potential confounders of the meat–cancer association, but neither proved to change the HR by greater than 10% and were not, therefore, included in the final models. Extremely fine control for smoking history and dosage was investigated, by constructing a 31-level variable using smoking status (never, former, current), time since quitting for former smokers, and smoking dose.

We examined models that were adjusted for energy by two different methods: the multivariate nutrient density method and the residual energy adjustment method [10]. Since both methods of energy adjustment gave similar results in our study, and because the interpretation of the multivariate nutrient density method applies to actual intakes as a percent of energy, which tends to be the units expressed for public health recommendations, we report the results from the multivariate nutrient density adjusted models.

Risks are reported for both sexes combined, unless there was a statistically significant interaction between meat intake and sex. Interactions were evaluated by including cross product terms in multivariate models. Furthermore, we conducted a lag analysis, excluding the first 2 y of follow-up, as well as various subanalyses to verify the stability of our risk estimates within subgroups of race, BMI, education, smoking status, physical activity level, and alcohol intake. The proportional hazards assumption was verified using a time interaction model. Tests for linear trend were calculated using the median value of each quintile. All reported *p-*values are two-sided. To test for heterogeneity, we used a chi-square test. To test for heterogeneity among the 21 cancer sites with 20 degrees of freedom, we first calculated the weighted average of the 21 beta coefficients from the Cox model, with weights being proportional to the inverse of the variances. Then we calculated the following chi-square statistic: 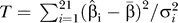 , where 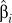 and 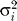 are the coefficient and its variance for each cancer, and β̄ is the weighted average of the beta coefficients. Likewise, to test for heterogeneity between subsites, such as colon versus rectum, we used the same chi-squared test given above, but using one degree of freedom. We also calculated population-attributable risks as an estimate of the percentage of cases that could be prevented if individuals adopted intake levels within the first quintile, given the assumption of a causal association between red or processed meat intake and cancer. All statistical analyses were carried out using Statistical Analytic Systems (SAS) software (SAS Institute).

## Results

During a mean follow-up of 6.8 y, 53,396 cancer diagnoses (36,907 male cases and 16,489 female cases) were ascertained. The mean energy–adjusted red meat intake in this cohort was 34.6 g/1,000 kcal (38.0 g/1,000 kcal in men and 29.5 g/1,000 kcal in women). The medians of extreme quintiles ranged from 9.8 to 62.7 g/1,000 kcal for red meat and 1.6 to 22.6 g/1,000 kcal for processed meat.

In general, those in the highest quintile of red meat intake tended to be slightly younger, less educated, less physically active, and less likely to consume fruits, vegetables, and alcohol than those in the lowest quintile. In contrast, those in the highest quintile of red meat intake were more likely to have a higher total energy intake, a higher BMI, and more likely to be a current smoker. Women in the highest quintile of red meat intake were also more likely to be married than those in the lowest quintile (Table 1).

**Table 1:**
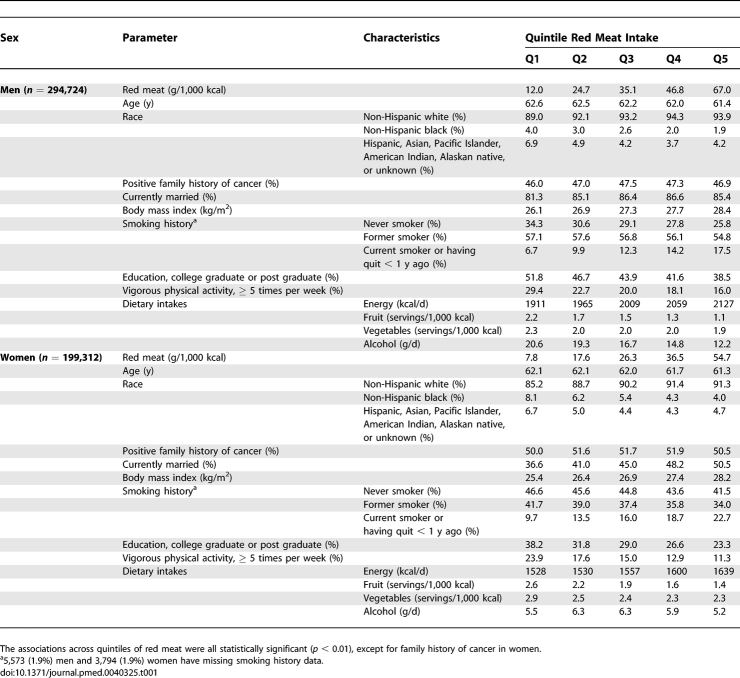
Means and Proportions for Baseline Characteristics of the NIH-AARP Diet and Health Study Cohort (*n* = 494,036) by Red Meat Quintile

### Red Meat

Individuals in the highest quintile of red meat intake, compared with those in lowest, had a statistically significant elevated risk of several malignancies (Table 2), including esophageal (HR = 1.51; 95% CI = 1.09–2.08; *p* for trend = 0.13), colorectal (HR = 1.24; 95% CI = 1.12–1.36; *p* for trend < 0.001, liver (HR = 1.61; 95% CI = 1.12–2.31; *p* for trend = 0.04), lung (HR = 1.20; 95% CI = 1.10–1.31; *p* for trend < 0.001), and borderline statistical significance for laryngeal cancer (HR = 1.43; 95% CI = 0.99–2.07; *p* for trend = 0.09). The positive association for red meat intake and colorectal cancer was due more to cancer of the rectum (*n* = 1,418; HR = 1.45; 95% CI = 1.20–1.75; *p* for trend < 0.001) than the colon (*n* = 3,689; HR = 1.17; 95% CI = 1.05–1.31; *p* for trend < 0.001), and this difference in risk was marginally statistically significant (*p* for heterogeneity = 0.06). Additional fine control for smoking did not alter the associations for cancers of the esophagus, colorectum, liver, lung, or larynx. In addition, the tests for interaction between smoking and both red meat (*p* interaction = 0.69) and processed meat (*p* interaction = 0.48) intake for lung cancer risk were not statistically significant. The population-attributable risks, representing the percentage of cases that could be prevented if individuals adopted red meat intake levels within the first quintile, were 33%, 9%, 35%, and 10% for esophageal, colorectal, liver, and lung cancer, respectively.

**Table 2:**
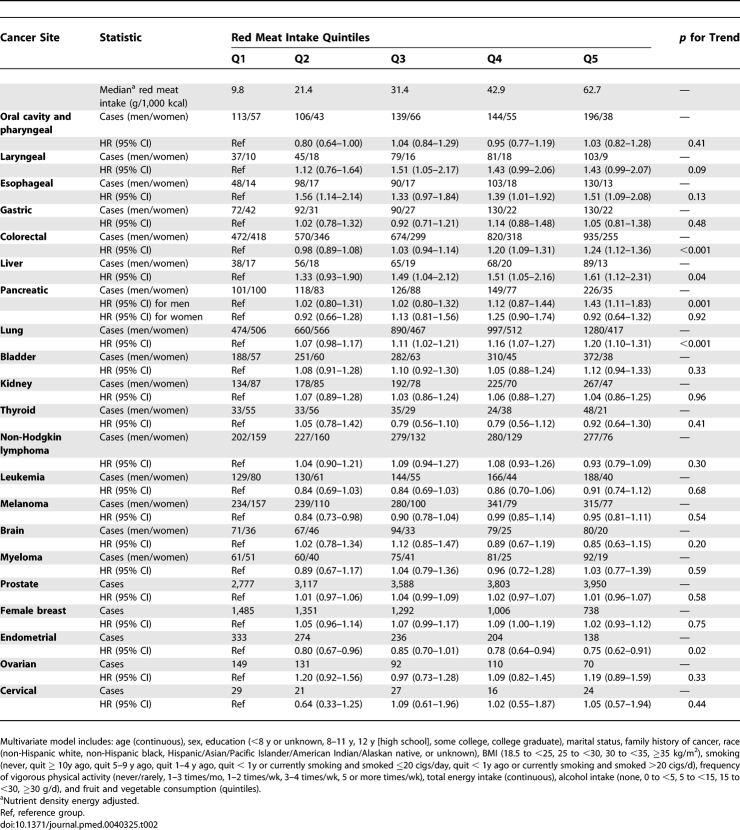
Multivariate HRs and 95% CIs (Both Sexes Combined) for Red Meat Intake and Cancer in the NIH-AARP Diet and Health Study

**Figure 1:**
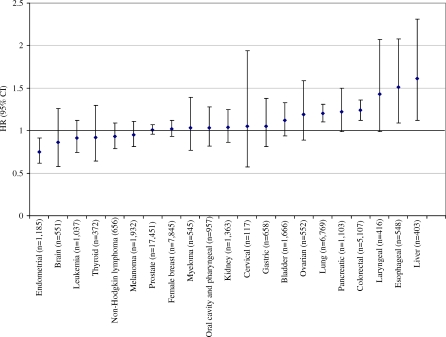
HRs and 95% CIs for the 5th Versus 1st Quintile of Red Meat Intake and Cancer Risk for Both Sexes Combined (Except for Sex-Specific Cancers)

Red meat intake was not associated with gastric or bladder cancer, leukemia, lymphoma, or melanoma. The associations between red meat and cancer are summarized in Figure 1, arranged by order of the magnitude of the risk; the figure also shows the null findings for sex-specific cancers, such as breast, ovarian, cervical, and prostate cancer. Unexpectedly, red meat intake was inversely associated with endometrial cancer (HR = 0.75; 95% CI = 0.62–0.91; *p* for trend = 0.02).

In further sex-specific analyses, red meat intake was positively associated with pancreatic cancer among men only (HR = 1.43; 95% CI = 1.11–1.83; *p* for trend = 0.001; *p* interaction by sex = 0.03); this risk was not attenuated by fine control for smoking. We observed no differences in risk by sex for other cancer sites.

### Processed Meat

Individuals in the highest quintile, compared with those in the lowest quintile, of processed meat intake were at elevated risk for colorectal (HR = 1.20; 95% CI = 1.09–1.32; *p* for trend < 0.001) and lung cancer (HR = 1.16; 95% CI = 1.06–1.26; *p* for trend = 0.001) (Table 3). In concordance with the red meat association, the risk observed for processed meat and colorectal cancer was slightly higher for rectal (HR = 1.24; 95% CI = 1.03–1.49; *p* for trend = 0.03) than colon cancer (HR = 1.18; 95% CI = 1.06–1.32; *p* for trend = 0.001), although this difference in risk was not statistically significant (*p* for heterogeneity = 0.67). Additional fine control for smoking did not alter the risk estimates for cancers of the colorectum or lung. Furthermore, borderline statistically significant increased risks for bladder cancer (HR = 1.16; 95% CI = 0.98–1.38; *p* for trend = 0.26) and myeloma (HR = 1.30; 95% CI = 0.98–1.71; *p* for trend = 0.01) were observed for those in the highest quintile of processed meat intake. The population-attributable risks, representing the proportion of cases potentially preventable if individuals adopted processed meat intake levels within the first quintile, were 10% for colorectal cancer and 9% for lung cancer.

**Table 3:**
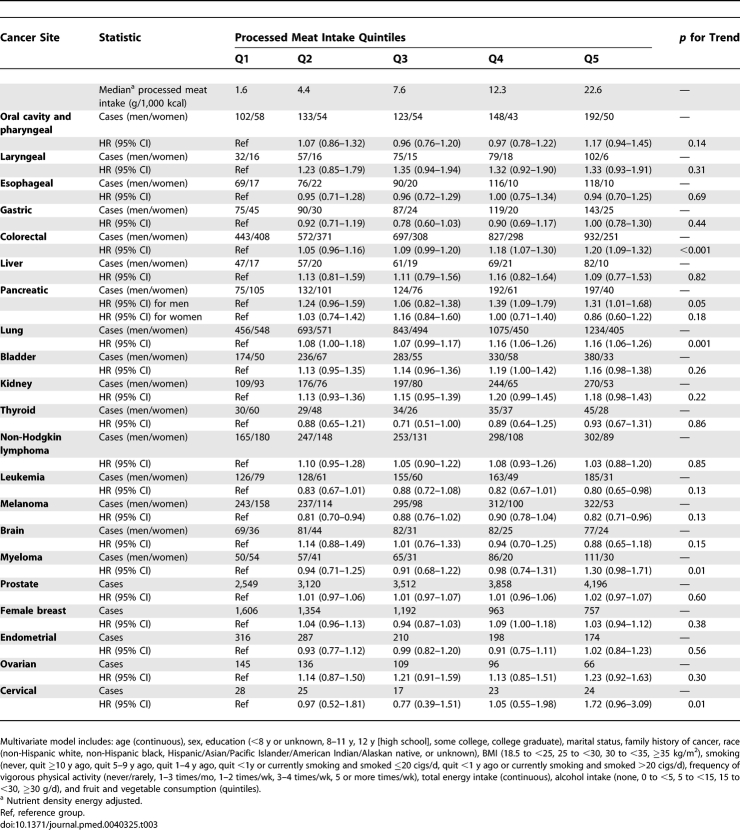
Multivariate HRs and 95% CIs (Both Sexes Combined) for Processed Meat Intake and Cancer in the NIH-AARP Diet and Health Study

**Figure 2:**
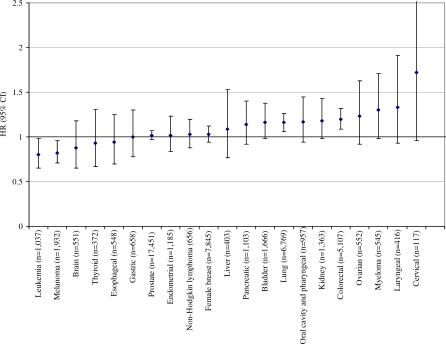
HRs and 95% CIs for the 5th Versus 1st Quintile of Processed Meat Intake and Cancer Risk for Both Sexes Combined (Except for Sex-Specific Cancers)

Surprisingly, both leukemia and melanoma were inversely associated with processed meat intake; the inverse association for leukemia was mainly for lymphocytic leukemia (*n* = 534; HR = 0.70; 95% CI = 0.52–0.93; *p* for trend = 0.05) and not myeloid and monocytic leukemia (*n* = 457; HR = 0.88; 95% CI = 0.64–1.20; *p* for trend = 0.73). The associations between processed meat intake and cancer risk are summarized in Figure 2, in order of risk magnitude.

Sex-specific analyses revealed a positive association for men in the highest quintile of processed meat consumption for pancreatic cancer (HR = 1.31; 95% CI = 1.01–1.68; *p* for trend = 0.05; *p* interaction = 0.01); this association was not attenuated by fine control for smoking. Furthermore, women in the highest quintile of processed meat had a borderline statistically significant elevated risk for cervical cancer (HR = 1.72; 95% CI = 0.96–3.09; *p* for trend = 0.01).

Further analyses did not reveal differences in risk associated with either red or processed meat intake for squamous versus adenocarcinoma of the esophagus, cardia versus non-cardia gastric cancer, and pre- versus post-menopausal breast cancer (unpublished data). However, we observed a suggestion of an elevated risk for advanced prostate cancer (*n* = 1,782), for both red meat (HR = 1.15; 95% CI = 0.98–1.36; *p* for trend = 0.21) and processed meat intake (HR = 1.22; 95% CI = 1.05–1.43; *p* for trend = 0.08), when comparing those in the highest with those in the lowest quintile of intake.

We conducted sensitivity analyses excluding processed meats from the red meat variable to determine whether the risks associated with red and processed meat are independent of each other. The removal of processed meats from the red meat variable reduced the median intake of red meat from 31.4 g/1,000 kcal to 23.6 g/1,000 kcal. The positive associations for red meat and cancer of the liver, esophagus, colorectum, and lung all remain when processed meats were removed from the red meat variable. Furthermore, the inverse association for red meat and endometrial cancer remained after removing processed meats.

In a lag analysis excluding the first two years of follow-up, both the positive and the inverse associations reported in this study remained. A stepwise addition of the covariates to a simple age- and sex-adjusted model showed that the effects of red and processed meat intake on cancer risk were attenuated the most by the addition of the smoking variable to the models. Subanalyses showed that there was no clear gradient in risk for colorectal or lung cancer across categories of education, BMI, physical activity, or alcohol intake. With regard to race, the increased risk observed for red and processed meat and cancer of the colorectum and lung was not evident for blacks, although this ethnicity only represented 3.8 and 4.0 percent of the cases for each cancer site, respectively, and therefore the confidence intervals were wide. The risks for lung cancer associated with both red and processed meat intake were apparent across all categories of smoking.

## Discussion

In this large, prospective investigation of red and processed meat intake in relation to cancer risk, we found elevated risks for colorectal and lung cancer with both meat types. Red, but not processed, meat intake was also associated with increased risk for cancer of the esophagus and liver. We observed borderline statistically significant elevated risks for advanced prostate cancer with both red and processed meat intake, for laryngeal cancer with red meat, and for bladder cancer and myeloma and with processed meat intake.

The cancer site most consistently associated with meat intake has been the colorectum. A recent meta-analysis of 15 prospective studies published through March, 2006, included approximately 7,500 cases, and reported elevated risks in the highest category of consumption of 28% for red meat and 20% for processed meat [9]. Our study included over 5,000 colorectal cancer cases, and it lends strong support to implicate red and processed meat as risk factors for this malignancy. Consistent with previous studies [9], we observed a stronger positive association for rectal than colon cancer.

The positive associations for both red and processed meat that we report for lung cancer were of similar magnitude to the findings for colorectal cancer. To date, our study includes the largest prospective analysis of meat intake and lung cancer risk. Previous case-control studies have reported elevated risks for lung cancer for those in the highest categories of red meat [17–19], fried red meat [8,19], well-done red meat [17], and processed meat intake [20]. Despite conducting analyses to show that very fine control of smoking history, using a 31-level variable, did not attenuate the lung cancer associations, there remains a potential issue of residual confounding by smoking, because it is such a strong risk factor for this disease.

We found a positive association between red meat intake specifically and cancers of the esophagus and liver, and a borderline significant positive association for laryngeal cancer. The first prospective study of meat intake and esophageal cancer was published recently; that study had only 65 cases and found a positive association for processed meat, but not red meat, with esophageal adenocarcinoma [21]. Our study suggests a threshold effect for red meat intake on esophageal cancer risk, beginning at a low level of intake, with no further increase in risk with higher intakes, as reflected in the *p*-trend (*p* = 0.13), although it is possible that the referent group had a smaller-than-expected cancer incidence by chance. Data on meat intake and cancers of the liver and larynx are limited, and our study is the first prospective investigation to report on these associations. Two case-control studies reported elevated risks for laryngeal cancer for those in the highest intake categories of red meat intake [22,23] and fried beef/veal [24].

In our study, those in the highest quintile of processed meat intake had borderline statistically significant elevated risks for myeloma, a malignancy that has not been well-studied for dietary associations, and bladder cancer. A study of two prospective cohorts combined [25], and one case-control study [26], both found elevated risks of bladder cancer for those in the highest categories of processed meat consumption, but another cohort study found no association [27].

Unexpectedly, we found an inverse association between red meat intake and endometrial cancer; this association was not attenuated by adjustment for known risk factors, such as body mass index or menopausal hormone therapy, or by fine control for smoking, which has been inversely associated with this malignancy [28]. Previous studies have reported null [29,30] or positive relations [31] between red meat and endometrial cancer. We also observed inverse associations between processed meat intake and leukemia and melanoma. In contrast to our findings, childhood leukemia has been positively associated with intake of processed meats in a case-control study [32].

Both red and processed meat intake were positively associated with pancreatic cancer in men, but not women. Red meat has been associated with pancreatic cancer in some [33,34], but not all [35–39] previous cohort studies, as has processed meat in one cohort [34] and several case-control studies [40–44]; although a sex-specific association has not been reported before. Although the association between pancreatic cancer and red or processed meat intake in men was unchanged by fine control for smoking, residual confounding by smoking is still possible.

Our study did not reveal an association between red or processed meat intake and gastric, prostate, or breast cancer, or non-Hodgkin lymphoma. In contrast to the positive relation between both red and processed meat intakes and noncardia gastric cancer in a large cohort in Europe [21], we found no differences in risk according to gastric anatomic subsite. The evidence for a positive association between meat intake and gastric cancer has been more consistent for processed meat than red meat, with elevated risks for processed meat in several case-control [45–52] and cohort studies [53–57], whereas red meat has been positively associated in some [45,46], but not all studies [47,58,59].

Previous studies of meat intake and prostate cancer are conflicting. Some studies have reported null findings [5,60–66], and others suggest positive associations [67–74]. Despite finding no association between red or processed meat intake and overall prostate cancer risk, we observed a suggestion of an elevated risk for advanced prostate cancer with both meat types. If the relation of meat intake to prostate cancer is confined to advanced disease, this could explain some of the inconsistencies in the literature as most previous studies have not specifically addressed advanced prostate cancer.

With regard to breast cancer, a pooled analysis of eight cohort studies found no association with red meat intake [75]; however, the two most recent prospective studies found positive associations for both red and processed meat [76], specifically for estrogen and progesterone receptor–positive breast cancers in premenopausal women [77]. Although breast cancer risk related to meat intake did not appear to differ by menopausal status in our study, we had very few premenopausal cases (*n* = 94) and lacked information on hormone receptor status for a large number of cases.

In agreement with our findings, the majority of studies of red meat and non-Hodgkin lymphoma have been null [78–85], although elevated risks were reported in some studies [86–88]; similarly, of nine studies investigating processed meat and non-Hodgkin lymphoma [79–82,84,86–89], only two found a positive association [81,89]. In contrast to our null findings, some case-control studies have reported positive associations for red or processed meat intake and cancer of the oral cavity, pharynx [90,91], kidney [92], ovaries [83], thyroid [83], and brain [93], although data for these cancer sites are limited.

Both red meat, regardless of processing procedure, and processed meat can be linked to carcinogenesis by different mechanisms; for example, they are both sources of saturated fat and iron, which have independently been associated with carcinogenesis. Associations between saturated fat and cancer are likely to be related to energy balance in general, whereas iron is thought to contribute to carcinogenesis specifically by generating free radicals and inducing oxidative stress [94]. Most recently, dietary fat was positively associated with breast cancer [95], and iron intake was positively associated with liver [96] and colorectal cancers [97].

Meat is also a source of several known mutagens, including *N*-nitroso compounds (NOCs), heterocyclic amines (HCAs), and polycyclic aromatic hydrocarbons (PAHs). Exposure to NOCs occurs from endogenous formation, which is directly related to red meat intake [98], and from exogenous exposure from nitrite-preserved meats, for example [1]. Red meat is a large source of readily available heme iron, which has been associated with increased endogenous NOC formation [99]. Human exposure to NOCs and subsequent cancer risk has not been studied extensively; although a Finnish cohort reported an increased risk of colorectal cancer with exogenous exposure to *N*-nitrosodimethylamine (from smoked and salted meats) [6]. In addition, NOC intake and excretion were significantly greater in an area within China considered as high-risk for esophageal cancer [100]. HCAs and PAHs, which are formed during high-temperature cooking of meat [101], dose-dependently generate DNA adducts [3] and tumors in rodents [2,102] in a wide variety of tissues and organs, with similarities between experimental animals and humans [103]. Epidemiologic studies with the capacity to estimate HCA and PAH intake from meat cooking information have found elevated risks of colorectal neoplasia [104–106], squamous cell esophageal cancer [107], as well as cancers of the lung [8], pancreas [4], and prostate [5].

With regard to the stronger relation of red and processed meat to rectal cancer than to colon cancer, there is variation in several characteristics along the large intestine; for example, the average crypt length [108], apoptotic index [109], and propensity to form *0* ^6^-methylguanine adducts[110] (pro-carcinogenic and a marker of NOC exposure) is greater toward the rectum than in the proximal colon. Furthermore, there is variation throughout the colorectum in bacterial enzymes [111], in the fermentation of short chain fatty acids [112], in the expression of metabolic enzymes [113], and in the concentration of fecal matter, in which potential carcinogens are concentrated toward the rectum.

Despite abundant biologic pathways linking meat intake to carcinogenesis at numerous anatomic sites, this is the first comprehensive and prospective analysis of meat intake in relation to a full range of malignancies. A particular strength of this study includes the large size of the cohort, which enabled us to investigate low-incidence cancers that have not previously been prospectively explored. Our findings are strictly generalizable to US whites over 50 y old, but may readily extend to other populations, because it is unlikely that the mechanisms relating meat to carcinogenesis differ quantitatively between our study population and those to whom our results do not strictly apply. An additional strength was that our study encompassed a wide range of reported meat intake, providing adequate statistical power to detect associations. Furthermore, recall bias and reverse causation were minimized by the assessment of diet prior to cancer diagnoses.

Potential limitations of this study include some degree of measurement error associated with the assessment of dietary and lifestyle variables. The FFQ used in this study was compared with two 24-h recall diaries in a subgroup of individuals from this cohort. The energy-adjusted correlation coefficients for protein and saturated fat, the two most relevant macronutrients for meat, were 0.43 and 0.50 for men and women, respectively, for protein, and 0.76 and 0.69 for men and women, respectively, for saturated fat [114]. These correlations compared very favorably to other commonly used FFQs that have correlations ranging from 0.29 to 0.61 in men and from 0.16 to 0.67 in women for protein intake and from 0.64 to 0.76 in men and from 0.59 to 0.76 in women for saturated fat intake [114]. Furthermore, the correlations for red meat assessed from the FFQ compared with two 24-h recall dairies were 0.62 for men and 0.70 for women [12]. Although some measurement error remains, the error associated with FFQs tends to result in attenuated risks [115], and we attempted to minimize this error by energy adjustment of the models [116]. With regard to nondietary covariates, reviews and meta-analyses have concluded that self-reported smoking behavior is accurate in most studies as assessed by plasma cotinine levels [117], and the correlations for physical activity assessment in questionnaires similar to that used in our study produce reasonable correlations [118]. Furthermore, self-reported height and weight is a reliable method of estimating BMI.

Some of the observed associations between meat intake and cancer risk in our study may be explained by exposure to HCAs and PAHs from meats cooked well-done by high-temperature cooking techniques; however, we lacked data on detailed cooking preferences from baseline. In addition, because we analyzed cancer incidence at multiple sites, some of our findings may have occurred by chance.

In conclusion, a diet high in red or processed meat was associated with an elevated risk of both colorectal and lung cancer; in addition, red meat was associated with an elevated risk of esophageal and liver cancer. A decrease in the consumption of red and processed meat could reduce the incidence of cancer at multiple sites.

## Supporting Information

Alternative Language Abstract S1 German Translation of Abstract by M. F. Leitzmann (21 KB DOC)

Text S1 Meat and Cancer STROBE Statement for Study (64 KB DOC)

## Abbreviations

AARP: formerly known as the American Association for Retired Persons
BMI: body mass index
CI: confidence interval
FFQ: food frequency questionnaire
HR: hazard ratio
HCA: heterocyclic amine
NAT: N-acetyltransferase
NOC: N-nitroso compound
PAH: polycyclic aromatic hydrocarbon
RR: relative risk
