# Supplementary material for: A Prospective Study of Red and Processed Meat Intake in Relation to Cancer Risk
Source: PLoS Med. 2007 Dec 11;4(12):e325. doi: 10.1371/journal.pmed.0040325 (PMC2121107; doi:10.1371/journal.pmed.0040325)
Supplement: Supplementary file 1 [file pmed.0040325.sd001.doc]

**A Prospective Study of Red and Processed Meat Intake in Relation to Cancer Risk**

Amanda J. Cross, PhD, Michael F. Leitzmann, MD, DrPH, Mitchell H. Gail, MD, PhD,

Albert R. Hollenbeck, PhD, Arthur Schatzkin, MD, DrPH, Rashmi Sinha, PhD

***Translation of abstract into German by Michael F. Leitzmann:***

**Hintergrund:** Studien belegen, das sowohl der Verzehr von rotem als auch von verarbeitetem Fleisch mit bestimmten Krebsarten in Verbindung steht. Es mangelt jedoch an prospektiven Daten bezüglich des Gesamtzusammenhangs zwischen Fleischkonsum und der Entwicklung einzelner Krebserkrankungen. Wir haben daher die Beziehungen zwischen dem Verzehr von rotem bzw. verarbeitetem Fleisch und den Risiken einer umfassenden Reihe von Krebserkrankungen untersucht. **Methoden und Ergebnisse:** Die NIH-AARP Diet and Health Study ist eine Kohorte von etwa 500.000 Menschen, die bei Studienbeginn in den Jahren 1995-1996 zwischen 50 und 71 Jahre alt waren. Die Fleischzufuhr der Teilnehmer wurde zu Studienbeginn mit einem semiquantitativen Ernährungsfragebogen ermittelt. Die Schätzung der relativen Risiken mit ihren entsprechenden 95% Konfidenzintervallen erfolgte durch Cox Proportional-Hazard-Regressionsmodelle. Während einer Follow-up Zeit von bis zu 8,2 Jahren wurden insgesamt 53.396 neuaufgetretene Krebsfälle erfasst. Verglichen mit der niedrigsten Quintile, zeigte die höchste Quintile des Verzehrs von rotem Fleisch statistisch signifikante, um 20-60% erhöhte relative Risiken für Karzinome des Ösophagus, des Kolorektums, der Leber, und der Lunge. In Bezug auf verarbeitetes Fleich zeigte sich zudem für die hoechste verglichen mit der niedrigsten Quintile der Zufuhr ein um 20% erhöhtes relatives Risiko für Karzinome des Kolorektums sowie der Lunge. **Schlussfolgerung:** Sowohl die Zufuhr von rotem als auch verarbeitetem Fleisch ist mit einem erhöhten Risiko des kolorektalen Karzinoms und des Bronchialkarzinoms verbunden. Die Zufuhr von rotem Fleisch zeigte zudem ein erhöhtes Risiko für das Ösophaguskarzinom und das Leberkarzinom.
