# Supplementary material for: A Prospective Study of Red and Processed Meat Intake in Relation to Cancer Risk
Source: PLoS Med. 2007 Dec 11;4(12):e325. doi: 10.1371/journal.pmed.0040325 (PMC2121107; doi:10.1371/journal.pmed.0040325)
Supplement: Supplementary file 2 [file pmed.0040325.sd002.doc]

**STROBE statement: Checklist of essential items Version 3 (Sept 2005) - *Cohort study***

| ***Section*** | ***Item*** | ***Checklist*** | ***Location in final version of manuscript*** |
| --- | --- | --- | --- |
| ***TITLE & ABSTRACT*** | ***1*** | *a)* Identify the article as a cohort study in the title or the abstract.  *b)* The abstract should be an informative and structured summary of the article, addressing key items in this checklist. | *1a) Line 5, page 2*  *1b) see overall abstract* |
| ***INTRODUCTION*** |  |  |  |
| ***Background / Rationale*** | ***2*** | Explain scientific background and rationale for the investigation being reported. | *See page 3* |
| ***Objectives*** | ***3*** | State specific objectives including any pre-specified hypotheses. | *Objective: see page 3, line 17 -20. Pre-specified hypotheses based on Page 3, paragraphs 1 and 2* |
| ***METHODS*** |  |  |  |
| ***Study design*** | ***4*** | Present key elements of study design. State purpose of original study, if article is one of several from an ongoing study. | *See page 4* |
| ***Setting*** | ***5*** | Describe setting, locations and dates defining periods of data collection. | *See page 4 and 5* |
| ***Participants*** | ***6*** | (a) Give inclusion and exclusion criteria, sources and methods of selection of participants.  (b) Give period and methods of follow-up. | *a) See page 5 (paragraph 2) and page 6 (paragraph 1)*  *b) See page 5, paragraph 2* |
| ***Variables of interest*** | ***7*** | List and clearly define all variables of interest indicating which are seen as outcomes, exposures, potential predictors, potential confounders or effect modifiers. | *Outcome definition: Page 5, line 17-22*  *Exposure variables: Page 4, line 22-page 5, line 5.*  *Confounders: page 6, line 16– page 7, line 6.* |
| ***Measurement*** | ***8*** | a)For each variable of interest give details of methods of assessment  b) If applicable, describe comparability of assessment methods across groups. | *Method of assessment: Page 4, paragraph 2.* |
| ***Bias*** | ***9*** | Describe any measures taken to address potential sources of bias. | *Page 16, line 16 we state that the ascertainment of diet prior to cancer diagnoses minimized recall bias as well as reverse causation – which itself was additionally addressed by a lag analysis (described page 7, line 9-12).* |
| ***Sample size*** | ***10*** | Describe rationale for study size, including practical and statistical considerations. | *The cohort was initiated to look at the effects of diet on health. Dietary-related risks tend to be modest (~1.2-1.3), and so the cohort purposely recruited enough individuals to result in sufficient power for dietary analyses. In the methods section of our manuscript we refer to the original study design (page 4, line8).* |
| ***Statistical methods*** | ***11*** | a)Describe all statistical methods including those to control for confounding.  (b) Describe how loss to follow-up and missing data were addressed.  (c) If applicable, describe methods for subgroup analyses and sensitivity analyses. | *a) See pages 6 and 7*  *b) see page 5, line 10-12. Those lost to follow-up in this cohort were very small (<5%) due to a variety of mailings to the participants. The overall cohort design and follow-up is referred to on page 4, line8. Missing data was also minimal at less than 3%.We will add the information on missing data to the manuscript (page 6, lines 18-20): “Missing data was minimal for this study; where appropriate, i.e. for smoking and body mass index (BMI), we created ‘missing’ categories but for others, such as family history of cancer, we set individuals missing this data to zero – no family history.”*  *c) see page 7, line 9-12* |
| ***Quantitative variables*** | ***12*** | (a) Explain how quantitative variables are analyzed e.g. which groupings are chosen, and why.  (b) Present results from continuous analyses as well as from grouped analyses, if appropriate. | *a) page 6, line 16*  *b) the continuous data does not add anything to the already dense tables in this manuscript. Furthermore, the continuous data forces linearity for all of the endpoints, which is an assumption we would prefer not to make.* |
| ***Funding*** | ***13*** | Give source of funding and role of funder(s) for the present study and, if applicable, the original study on which the present article is based. | *See page 18, lines 5-22* |
| ***RESULTS*** |  |  |  |
| ***Participants*** | ***14*** | (a) Report the numbers of individuals at each stage of the study, e.g. numbers potentially eligible, examined for eligibility, confirmed eligible, included in the study, completing follow up, and analysed.  (b) Give reasons for non-participation at each stage.  (c) A flow diagram is recommended.  (d) Report dates defining period of recruitment. | *a) page 6, paragraph 1 and page 8, lines 1-2.*  *b) given in paragraph 1 of page 6.*  *c) we do not feel a flow diagram is necessary for the description of our population.*  *d) see page 4, lines 3-9 and page 5, lines 10-12.* |
| ***Descriptive data*** | ***15*** | (a) Give characteristics of study participants (e.g. demographic, clinical, social) and information on exposures and potential confounders  (b) Indicate for each variable of interest the completeness of the data.  (c) Summarize average and total amount of follow up and dates defining follow up. | *a) see page 4, lines 3-5 and page 8, lines 2-12, as well as Table 1.*  *b) page 4, lines 8-9, refer to the original description of the cohort, during which respondents were removed if they had skipped large portions of their food frequency questionnaire, or had >10 recording errors, or <10 foods reported in usual diet. We also removed those with extreme (unrealistic) energy intake – please see page 6, lines 8-11.*  *c) see page 5, line 10 – 16 and page 8, line 1.* |
| ***Outcome data*** | ***16*** | Report numbers of outcome events or summary measures over time. | *See page 8, lines 1-2, and tables 1, 2 and 3, as well as Figures 1 and 2.* |
| ***Main results*** | ***17*** | (a) Give unadjusted and confounder adjusted measures of association and their precision (e.g. 95% confidence intervals). Make clear which confounders were adjusted for and on what grounds they were included and others were not.  (b) For comparisons using categories derived from quantitative variables, report the range of values or median value in each group.  (c) Translate relative measures into absolute differences, for a meaningful risk period that does not extend beyond the range of the data.  (d) Report results standardized to confounder and modifier distributions for realistic target populations. | *a) We addressed the issue of presenting unadjusted risk estimates in our cover letter to the senior editor. This manuscript already contains very dense tables, and we feel that additionally including all of the unadjusted risk estimates will cause too much clutter. However, if the editors would prefer the unadjusted risk estimated to be included, we would be happy to do so. Information on confounders is given on page 6, lines 16-2.*  *b) See page 8, lines 2-5, as well as Tables 2 and 3.*  *c) We will add the following to the methods section (page 7, line 21): “We also calculated population attributable risks as an estimate of the percentage of cases that could be prevented if individuals adopted intake levels within the first quintile, given the assumption of a causal association between red or processed meat intake and cancer.”*  *We will add the following sentence to the results (page 9, lines 8-11 ): “The population attributable risks, representing the percentage of cases that could be prevented if individuals adopted red meat intake levels within the first quintile, were 33%, 9%, 35% and 10% for esophageal, colorectal, liver and lung cancer, respectively*.*” And page 10, lines 11-14: “The population attributable risks, representing the proportion of cases potentially preventable if individuals adopted processed meat intake levels within the first quintile, were 10% for colorectal cancer and 9% for lung cancer.”*  *d) The population we have studied is a realistic target population, since the cohort recruited from 8 different states from all over the US. Tables 2 and 3 and Figures 1 and 2 report results adjusted for confounders. We have added a comment on generalizability of our findings as per item 22 listed below.* |
| ***Other analyses*** | ***18*** | Report any other analyses performed, e.g. subgroup analyses and sensitivity analyses. | *See page 9, lines 11-14, page 10, lines 10 to page 11, line 12.* |
| ***DISCUSSION*** |  |  |  |
| ***Key findings*** | ***19*** | Summarize key results with reference to study hypotheses. | *See page 11, lines 15-20.* |
| ***Limitations*** | ***20*** | (a) Discuss limitations of the study, taking into account sources of potential bias or imprecision, and problems that could arise from multiplicity of analyses, exposures and outcomes. Discuss both direction and magnitude of any potential bias.  (b) Consider that the discussion of limitations should not be used as a substitute for quantitative sensitivity analyses. | *a) See page 16, line 18 to page 17, line 16.* |
| ***Generalizability*** | ***21*** | Discuss the generalizability (external validity) of the study findings. | *We will add the following (page16, line 19): “Our findings are strictly generalizable to U.S. Caucasians over 50 years old, but may readily extend to other populations since it is unlikely that the mechanisms relating meat to carcinogenesis differ quantitatively between our study population and those to whom our results do not strictly apply.”* |
| ***Interpretation*** | ***22*** | Give a cautious overall interpretation of the results in the context of current evidence and study limitations, paying attention to alternative interpretations. | *Please see page16, line 15 to page 18, line 2.* |
